# Scarlet Fever

**Published:** 1881-07

**Authors:** 


### Editorial.

### SCARLET FEVER.

No small amount of excitement and alarm has recently been occasioned by the few cases of scarlatina that was in our midst but a short time since. Accounts of the so-called prevalence of this terrible disease exercised, more or less, the minds of strangers at a distance, to whom rumors of an *epidemic* in Atlanta bore a special anxiety, as many anticipated attending the great Cotton Exposition that has been arranged to take place in this city. As is usual in such cases, the slightest rumor is sufficient to kindle the fiercest flame, and the imagination wrought upon by ignorant fear constructs a wide-spread and devastating epidemic out of a few isolated sporadic cases.

This tendency of human nature is universal, and efforts directed to allaying the confusion incident to these epidemical alarms often result in rendering "confusion worse confounded." Other ills attend the sporadic appearance of a malignant disease, one of which is the proneness to pronounce each and every cotemporaneous disease as a more or less defined form of the malignant trouble. It would seem that each one aspires to present plausible indications of the continuance of the supposed epidemic.

This, in its last analysis, is pure demagogueism, mixed possibly with slight indifference to results.

Very few epidemic diseases have a list of symptoms peculiar to themselves. Many of their symptoms, and much of their morbid anatomy, characterize other diseases that may be benign in their disposition, or at least decidedly different in their pathological details.

For these reasons, at least, we should hesitate some time before we pronounce upon the malignant character of a disease, and especially so upon such an exigency as a community alarmed and demoralized over an anticipated epidemic.

Scarlet fever which was the scare-crow to so many in the recent "plague" excitement does not so certainly possess the power or property of contagion that is claimed by most writers on the subject. In fact, the pronounced cases that we have had in our midst go far to illustrate a view contrary to the contagion theory. In the neighborhood of the Perkins family, where the most fatal outburst of the malady raged, a complete immunity from it ravages existed. No other case was noticed in that section, which certainly would not have been the case if the disease is so highly contagious as is claimed for it.

Again, there is no evidence that the disease was transmitted by any of the many visitors who called on the Perkins family during the prevalence of the fever there.

In conclusion we will not hesitate to affirm, as our honest opinion, that no city in the Union is so safe against epidemics as Atlanta. We might go further and say that no other city can claim, successfully, a superiority over us in the way of health and freedom from epidemic or endemic influences.

